# β-elemene suppresses Warburg effect in NCI-H1650 non-small-cell lung cancer cells by regulating the miR-301a-3p/AMPKα axis

**DOI:** 10.1042/BSR20194389

**Published:** 2020-06-18

**Authors:** Lin Li, Dongkai Zhao, Guangyu Cheng, Qingjie Li, Yunjie Chu, Hongbo Chu, Yunlu Ding, Chikun Li

**Affiliations:** 1 Health Care Department, The Affiliated Hospital to Changchun University of Chinese Medicine, Changchun 130021, China; 2 Department of Pulmonary Disease, Third Clinical Hospital Affiliated to Changchun University of Traditional Chinese Medicine, Changchun 130000, China; 3 The Research Center of The Affiliated Hospital to Changchun University of Chinese Medicine, Changchun 130021, China; 4 Massage Department, The Affiliated Hospital to Changchun University of Chinese Medicine, Changchun 130021, China

**Keywords:** β-elemene, AMPKα, MiR-301a-3p, Non-small-cell lung cancer, Warburg effect

## Abstract

β-elemene has been evidenced to suppress the development of numerous cancers including lung cancer. Previous research has found that in A549 cells, β-elemene increased the expression of adenosine monophosphate-activated protein kinase (AMPK) α (AMPKα), which negatively regulates the Warburg effect. Bioinformatics predicted that binding sites exist between AMPKα and miR-301a-3p, an miRNA that has shown oncogenic function in many cancers. The aim of this work was to investigate the effect of β-elemene on the Warburg effect in non-small-cell lung cancer (NSCLC) cells and its mechanism. Herein, the expression of miR-301a-3p was evaluated in NSCLC cells. Then, miR-301a-3p was overexpressed or silenced by mimics or inhibitors, respectively, followed by treatment with AMPK agonists or antagonists. NSCLC cells subjected to miR-301a-3p overexpression or inhibition were further treated with β-elemene. The results demonstrated that AMPKα was targeted and negatively regulated by miR-301a-3p. AMPKα agonists attenuated the Warburg effect in NSCLC cells induced by miR-301a-3p, as evidenced by the decrease in glucose level, lactic acid level, and expression of metabolism-related enzymes (glucose transporter 1 (GLUT1), hexokinase 1 (HK1), and lactate dehydrogenase A (LDHA)). Additionally, β-elemene suppressed the expression of miR-301a-3p, enhanced that of AMPKα, and inhibited the Warburg effect in NSCLC cells. The results indicated that β-elemene attenuates the Warburg effect in NSCLC cells, possibly by mediating the miR-301a-3p/AMPKα axis.

## Introduction

Lung cancer is the most prevalent tumor worldwide, and approximately 85% of lung cancer cases are classified as non-small-cell lung cancer (NSCLC) [1]. In 2018, there were 2093876 new cases of lung cancer, accounting for 11.6% of the total number of cancer cases and 1761007 cancer-related deaths worldwide [2]. Despite substantial effort in understanding the underlying mechanism and the development of interventional therapy, the 5-year survival rate remains low because of the complex pathogenesis, difficulty in early diagnosis, and metastasis [3]. Thus, it is necessary to develop efficient therapeutic methods for the treatment of NSCLC.

Cancer cells preferentially use glucose for energy via glycolysis, even under aerobic conditions, a phenomenon identified as the ‘Warburg effect’ [4]. This phenomenon is associated with the expression of oncogenes, changes in the tumor microenvironment, and abnormal expression of glycometabolic enzymes, providing an advantage for cancer cell growth. Adenosine monophosphate-activated protein kinase (AMPK) is an important regulator of glucose uptake and energy balance and is a central component in the signal transduction network. Once activated, AMPK can phosphorylate key proteins through multiple pathways, affecting intracellular glycolysis and mitochondrial homeostasis [5]. Faubert et al. found that AMPKα is a negative regulator of the Warburg effect and inhibited the tumorigenic process *in vivo* [6]. Zhao et al. suggested that the activation of phosphorylated (p)-AMPK inhibited the growth of A549 NSCLC cells [7], and AMPK was involved in the regulation of the Warburg effect in NSCLC cells [8]. The regulatory mechanism of AMPK, which is not related to genomic changes, may also affect its activity in cancer, and one possible mechanism is regulation by miRNA [9]. As a carcinogenic factor, miR-301a-3p plays an important role in the occurrence and development of pancreatic [10], breast [11], colorectal [12], and other cancers. It has been reported that the expression of miR-301a-3p was abnormally high in lung cancer tissues [13], and bioinformatics analysis predicted that binding sites exist between miR-301a-3p and AMPK. However, whether the regulatory effect of AMPK on the Warburg effect in NSCLC cells is associated with miR-301a-3p remains unclear.

β-elemene is a major bioactive sesquiterpenoid extracted from *Curcuma wenyujin*, a Chinese medicinal herb [1], and has been demonstrated to suppress the development of numerous cancers, including lung [14], breast [15], and bladder cancer [16] by modulating multiple signaling pathways. Our preliminary studies indicated that β-elemene increased the expression of AMPKα in A549 cells, but whether the mechanism underlying the effect of β-elemene is mediated by miR-301a-3p remains to be investigated.

The aim of the current study was to investigate the effect of β-elemene on the Warburg effect in NSCLC cells and whether its underlying mechanism is related to the miR-301a-3p/AMPK axis. On this basis, we first investigated the effect of miR-301a-3p on the Warburg effect and AMPK activity in NSCLC cells. Then, the effect of β-elemene on the Warburg effect in NSCLC cells and its relationship with miR-301a-3p/AMPK axis were investigated.

## Materials and methods

### Experiment protocol

Human NSCLC cell lines A549 and NCI-H1650 (purchased from Shanghai Institutes for Biological Sciences, Chinese Academy of Science) and the normal human lung epithelial cell line BEAS-2B (purchased from the Cell Bank of Wuhan University) were subjected to quantitative reverse-transcription polymerase chain reaction (qRT-PCR) to detect miR-301a-3p expression level. Then, miR-301a-3p was overexpressed or inhibited in NCI-H1650 cells by miR-301a-3p mimics or inhibitors at 37°C for 24 h (Ribobio Co. Ltd, Guangzhou, China), respectively. Cell viability, AMPKα expression, and glucose metabolism-related factors were evaluated to investigate the effect of miR-301a-3p on AMPKα expression and the Warburg effect in NCI-H1650 cells.

Subsequently, NCI-H1650 cells wherein miR-301a-3p was overexpressed or inhibited were treated with an AMPK agonist (AICAR, 500 μl, Selleck, Shanghai, China) or antagonist (BML-275, 5 μl, Selleck) at 37°C for 24 h, respectively. The expression of miR-301a-3p, AMPKα1, and glucose metabolism-related factors were analyzed to explore whether AMPKα influences the effect of miR-301a-3p on the Warburg effect in NCI-H1650 cells.

Finally, both miR-301a-3p-overexpressing and miR-301a-3p-silenced NCI-H1650 cells were treated with β-elemene (50 μg/ml) at 37°C for 24 h and the expression of miR-301a-3p, AMPKα1, and glucose metabolism-related factors was analyzed to investigate the mechanism of β-elemene on NCI-H1650 cells.

### Cell culture

NCI-H1650 cells were cultured in RPMI-1640 medium (HyClone, Logan, U.S.A.) supplemented with 10% fetal bovine serum (FBS, Gibco, Gibco BRL, Gaithersburg, MD, U.S.A.). A549 cells were cultured in Ham’s F-12K medium (Gibco) supplemented with 10% FBS. BEAS-2B cells were cultured in Bronchial Epithelial Cell Growth Medium (Lonza, Basel, Switzerland). All cells were maintained in an atmosphere containing 5% CO_2_ at 37°C.

### qRT-PCR

Total RNA was extracted using TRIzol (Ambion, Texas, U.S.A.) and DNA was eliminated using DNase1 (Fermentas, Canada). The obtained RNA was reverse-transcribed into cDNA using the M-MLV kit (TaKaRa, Dalian, China) and amplified using the SYBR Green PCR kit (KAPA Biosystems, U.S.A.) according to the manufacturer’s instructions. The sequences were: miR-301a-3p forward: 5′-GGGCAGTGCAATAGTATT-3′, reverse: 5′-AACTGGTGTCGTGGAGTCGGC-3′; U6 forward: 5′-CTCGCTTCGGCAGCACA-3′, reverse: 5′-AACGCTTCACGAATTTGCGT-3′; AMPKα1 forward: 5′-AAAAGAAAGTCGGCGT-3′, reverse: 5′-GCATAGTTGGGTGAGC-3′; GAPDH forward: 5′-CCACTCCTCCACCTTTG-3′, reverse: 5′-CACCACCCTGTTGCTGT-3′. GAPDH and U6 served as endogenous controls of AMPKα1 and miR-301a-3p, respectively. The data were analyzed using the 2^−ΔΔ*C*_t_^ method [17].

### Cell counting kit-8 analysis

NCI-H1650 cells were harvested at the logarithmic growth phase and cultured in a 96-well plate at 5 × 10^3^ cells/well at 37°C in 5% CO_2_ overnight. After treatment for 24, 48, or 72 h, the cells were incubated with 10 μl of cell counting kit (CCK)-8 (CCK-8) solution for 4 h and the absorbance was analyzed using a microplate reader (all from Life Science Co., Ltd., Hangzhou, China) at 450 nm.

### Flow cytometry

Apoptosis was detected using the Annexin V-phycoerythrin (PE)/7-aminoactinomycin D (7-AAD) detection kit (BD, Shanghai, China). Cells (∼1 × 10^6^) were centrifuged at 4°C at 1000×***g*** for 5 min and resuspended in 1 ml of phosphate-buffered saline (Bioswamp, Wuhan, China). After centrifugation at 4°C at 1000×***g*** for 5 min, the cells were resuspended in 100 μl of binding buffer, followed by the addition of 5 μl of Annexin V-PE and 5 μl of 7-AAD. The cells were maintained at room temperature in the dark for 15 min. After the addition of 400 μl of binding buffer, the cells were subjected to flow cytometry (ACEA Biosciences, San Diego, CA, U.S.A.).

### Biochemical analysis

Glucose levels (Shanghai Rongsheng Biological Pharmaceutical Co., Ltd., Shanghai, China), and lactic acid content (Nanjing Jiancheng Bioengineering Institute) were evaluated using respective assay kits according to the manufacturer’s instructions.

### Western blot analysis

Total proteins were extracted from harvested cells using radioimmunoprecipitation assay lysis buffer (Bioswamp) and quantified using a bicinchoninic acid kit (Bioswamp) according to the manufacturer’s instructions. Proteins (20 μg) were separated and transferred on to polyvinylidene fluoride membranes (Millipore, MA, U.S.A.), followed by blocking with skim milk. The membranes were then incubated with primary antibodies against AMPKα1 (Bioswamp, PAB30970, 1:1000), p-AMPKα1 (Bioswamp, PAB36316-P, 1:1000), glucose transporter 1 (GLUT1, Bioswamp, MAB37348, 1:1000), hexokinase 1 (HK1, Bioswamp, MAB37234, 1:1000), lactate dehydrogenase A (LDHA, Bioswamp, PAB30703, 1:1000), and GAPDH (Bioswamp, PAB36269, 1:1000) overnight at 4°C, followed by incubation with goat anti-rabbit IgG secondary antibodies (Bioswamp, SAB43714, 1:20000) for 1 h at room temperature. GAPDH served as the internal reference.

### Dual luciferase reporter assay

The 3′-untranslated region (UTR) of AMPKα, with wild-type (WT) or mutant (MUT) binding sites for miR-301a-3p, was amplified and cloned into the pmirGLO vector (Addgen, Cambridge, MA, U.S.A.) to form pmirGLO-WT-AMPKα-3′ UTR or pmirGLO-MUT-AMPKα-3′ UTR plasmids. The plasmids and miR-301a-3p were then co-transfected into HEK293T cells using Lipofectamine™ 2000 (Invitrogen, Carlsbad, CA, U.S.A.). Luciferase activity was measured using the dual-luciferase reporter assay (Genecopoeia).

### Statistical analysis

Data are presented as the mean ± standard deviation (SD). Differences between more than two groups were analyzed using one-way analysis of variance followed by least significant difference or Dunnett’s T3 test. *P*<0.05 was considered to be statistically significant.

## Results

### MiR-301a-3p promotes NCI-H1650 cell proliferation and Warburg effect

**Figure 1:**
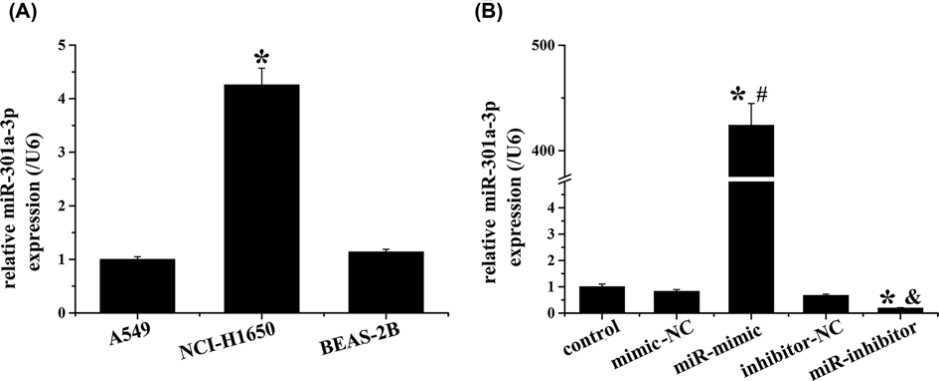
Relatively high expression of miR-301a-3p in NCI-H1650 cells (**A**) Relative expression of miR-301a-3p in A549, NCI-H1650, and BEAS-2B cells were detected using qRT-PCR. (**B**) Relative expression of miR-301a-3p in NCI-H1650 cells after transfection was detected using qRT-PCR. MiR-301a-3p was higher expressed in NCI-H1650 cells than others and miR-301a-3p expression in NCI-H1650 cells was overexpressed or inhibited after miR-301a-3p mimic or inhibitor transfection. Data represent the mean ± SD (*n*=3). *P<0.05 vs BEAS-2B in (A); *P<0.05 vs control, ^#^*P*<0.05 vs mimic-NC, ^&^*P*<0.05 vs inhibitor-NC in (B).

**Figure 2:**
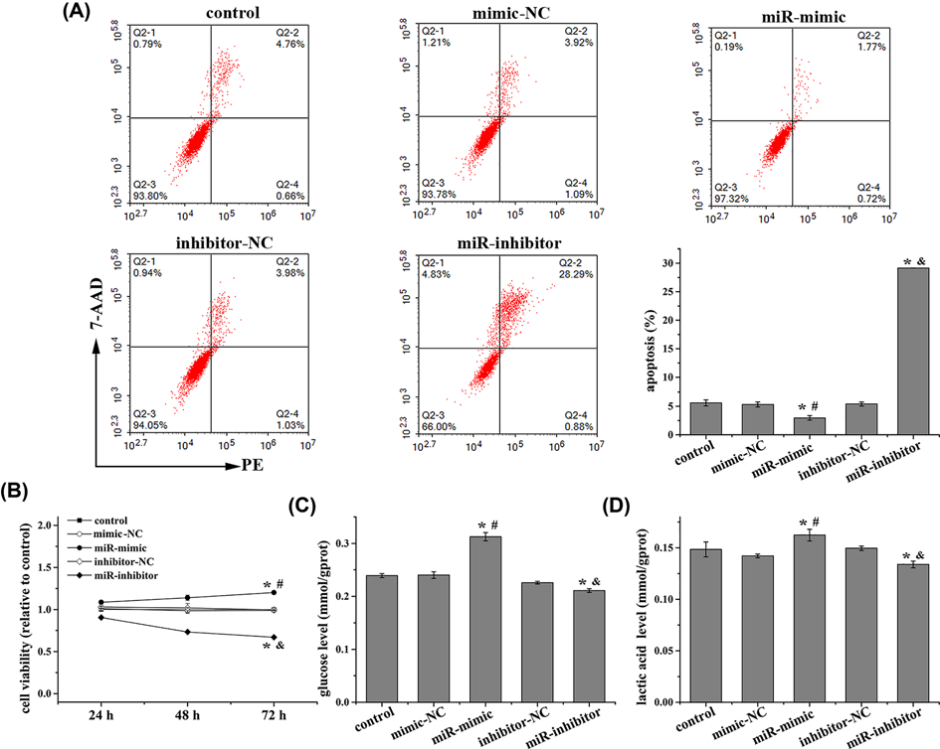
MiR-301a-3p promotes proliferation of NCI-H1650 cells and the Warburg effect (**A**) Apoptosis rate was detected using flow cytometry, (**B**) cell viability was detected using MTT, (**C**) glucose level, and (**D**) lactic acid level were detected using corresponding kits in NCI-H1650 cells after transfection. MiR-301a-3p overexpression decreases apoptosis rate, increases viability, glucose and lactic acid levels in NCI-H1650 cells. Data represent the mean ± SD (*n*=3). *P<0.05 vs control, ^#^*P*<0.05 vs mimic-NC, ^&^*P*<0.05 vs inhibitor-NC.

**Figure 3:**
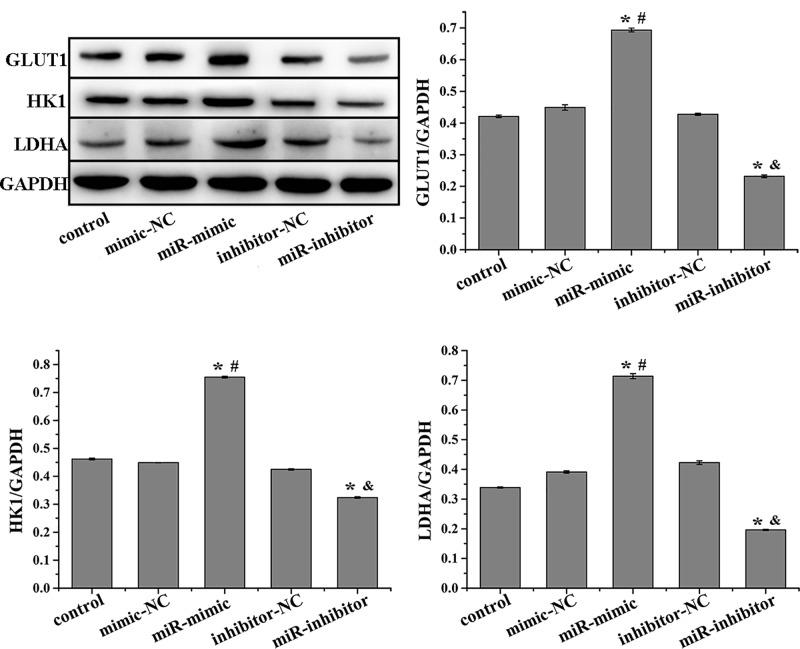
Western blot was performed to detect the protein expression of GLUT1, HK1, and LDHA in NCI-H1650 cells after transfection MiR-301a-3p overexpression increases the expression of GLUT1, HK1, and LDHA in NCI-H1650 cells. Data represent the mean ± SD (*n*=3). *P<0.05 vs control, ^#^*P*<0.05 vs mimic-NC, ^&^*P*<0.05 vs inhibitor-NC.

As shown in Figure 1A, the expression of miR-301a-3p was significantly higher in NCI-H1650 cells than those in normal lung epithelial cells (BEAS-2B) and A549 cells. Thus, the NCI-H1650 cell line was selected to investigate the effect of miR-301a-3p on the proliferation of NSCLC cells and the Warburg effect. The expression of miR-301a-3p in NCI-H1650 cells was enhanced and suppressed by miR-301a-3p mimics and inhibitors, respectively (Figure 1B). The apoptosis of NCI-H1650 cells was inhibited by miR-301a-3p overexpression but increased by miR-301a-3p inhibition (Figure 2A). Additionally, miR-301a-3p overexpression increased cell viability and enhanced the Warburg effect by increasing the levels of glucose and lactic acid and the expression of metabolism-related enzymes involved in the Warburg effect (GLUT1, HK1, and LDHA), whereas miR-301a-3p inhibitors had the opposite effect (Figures 2B–D and 3). Overall, miR-301a-3p enhanced the proliferation of NCI-H1650 cells and the Warburg effect.

### MiR-301a-3p promotes Warburg effect in NCI-H1650 cells by regulating AMPKα expression

**Figure 4:**
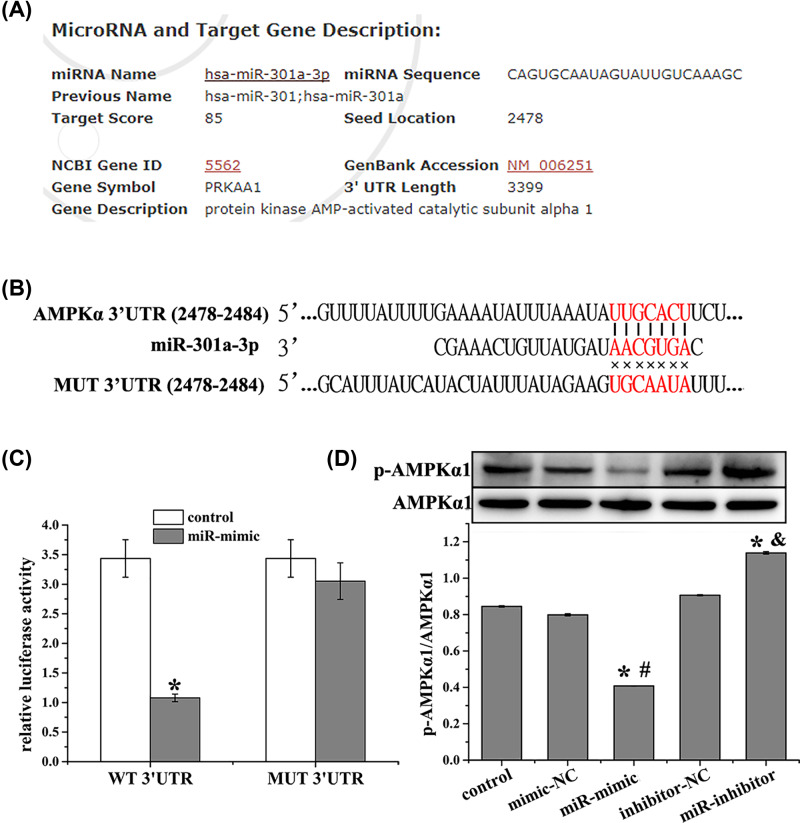
AMPKα is targeted by miR-301a-3p (**A**) AMPKα is predicated to be a target of miR-301a-3p using miRDB database. (**B**) Binding sites between AMPKα and miR-301a-3p were analyzed using TargetScan database. (**C**) Relative luciferase activity of the AMPKα-WT and AMPKα-MUT reporter plasmids in NCI-H1650 cells after transfection with miR-301a-3p mimics. (**D**) Relative protein expression of p-AMPKα in NCI-H1650 cells after transfection. AMPKα is revealed to be a target of miR-301a-3p and miR-301a-3p overexpression inhibits the phosphorylation level of AMPKα. Data represent the mean ± SD (*n*=3). *P<0.05 vs control, ^#^*P*<0.05 vs mimic-NC, ^&^*P*<0.05 vs inhibitor-NC.

**Figure 5:**
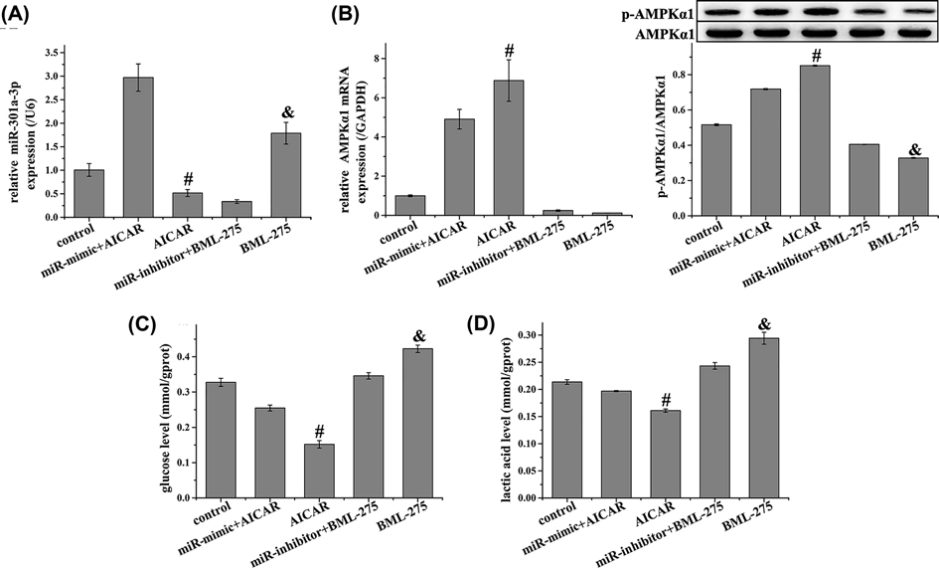
AMPK agonist inhibited the expression of miR-301a-3p and Warburg effect after it was promoted by miR-301a-3p mimic (**A**) Relative expression of miR-301a-3p was detected using qRT-PCR, (**B**) relative mRNA and protein expression of AMPKα1 were detected using qRT-PCR and Western blot, respectively, (**C**) glucose level was detected using the corresponding kit, and (**D**) lactic acid level was detected using the corresponding kit in NCI-H1650 cells. AMPK agonist inhibited the expression of miR-301a-3p, glucose level, and lactic acid level after they were promoted by miR-301a-3p mimic. Data represent the mean ± SD (*n*=3). ^#^*P*<0.05 vs miR-mimic + AICAR, ^&^*P*<0.05 vs miR-inhibitor + BML-275.

AMPKα was predicted to be a target of miR-301a-3p using the miRDB database (http://www.mirdb.org/cgi-bin/target_detail.cgi?targetID=1075038, Figure 4A). This was further confirmed by TargetScan analysis (http://www.targetscan.org/vert_72/, Figure 4B) and dual-luciferase reporter assay, which demonstrated that miR-301a-3p overexpression suppressed the luciferase activity of AMPKα-WT (Figure 4C). We further evaluated the expression of AMPKα1 in NCI-H1650 cells after miR-301a-3p was overexpressed or inhibited. MiR-301a-3p overexpression down-regulated AMPKα1 expression compared with that of the control group, while miR-301a-3p inhibition up-regulated AMPKα1 expression (Figure 4D). Furthermore, the AMPK agonist AICAR decreased the level of miR-301a-3p after it was up-regulated by miR-301a-3p mimics, and enhanced AMPK expression after it was down-regulated by miR-301a-3p mimics (Figure 5A,B). In addition, AICAR reduced the levels of glucose and lactic acid after they were increased by miR-301a-3p mimics (Figure 5C,D). The AMPK antagonist BML-275 showed the opposite function as that of AICAR. These results demonstrated that AMPK is involved in the regulation of the Warburg effect by miR-301a-3p in NCI-H1650 cells.

### β-elemene inhibits the Warburg effect in NCI-H1650 cells by regulating miR-301a-3p/AMPKα axis

**Figure 6:**
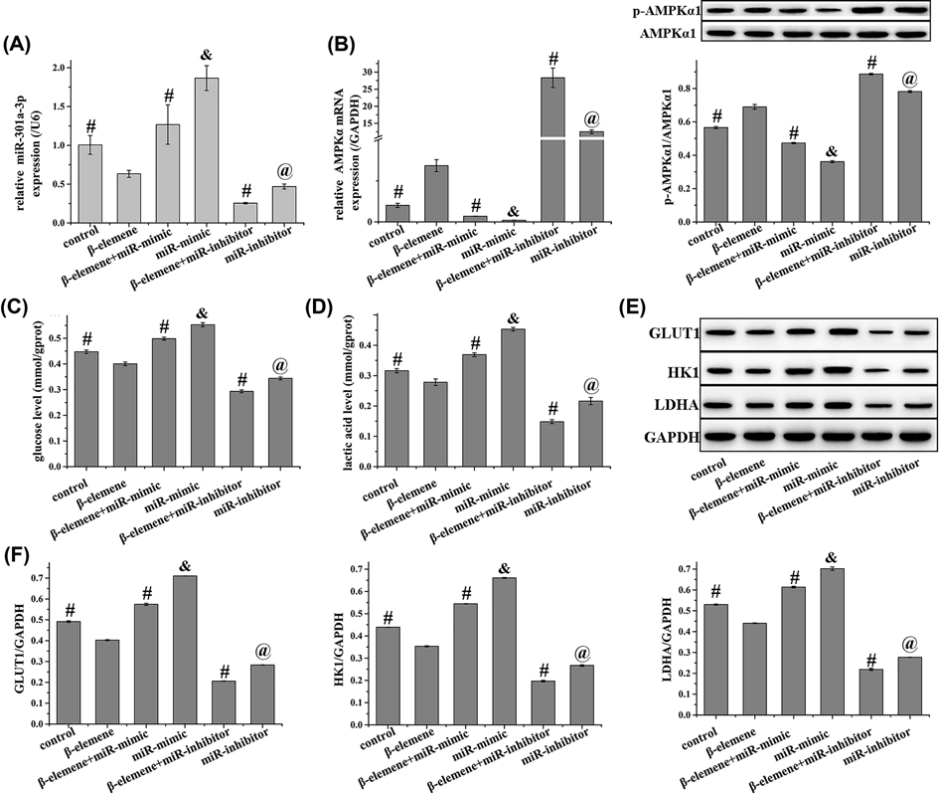
β-elemene inhibits the Warburg effect in NCI-H1650 cells by regulating miR-301a-3p/AMPKα axis (**A**) Relative expression of miR-301a-3p were detected using qRT-PCR, (**B**) relative mRNA (detection using qRT-PCR) and protein expression (detection using Western blot) of (p)-AMPKα1, (**C**) glucose level was detected using the corresponding kit, (**D**) lactic acid level was detected using the corresponding kit, (**E**) relative protein expression of GLUT1, HK1, and LDHA were detected using Western blot in NCI-H1650 cells, and (**F**) quantified from (E). β-elemene inhibits the expression of miR-301a-3p, GLUT1, HK1, LDHA, the levels of glucose and lactic acid and enhances the expression of AMPKα in NCI-H1650 cells, which are reversed by miR-301a-3p mimic. Data represent the mean ± SD (*n*=3). ^#^*P*<0.05 vs β-elemene, ^&^*P*<0.05 vs β-elemene + miR-mimic, ^@^*P*<0.05 vs β-elemene + miR-inhibitor.

As shown in Figure 6, compared with control cells and those treated with miR-mimics or miR-inhibitors, additional β-elemene treatment down-regulated miR-301a-3p expression and up-regulated that of AMPKα. Furthermore, β-elemene attenuated the Warburg effect in NCI-H1650 cells, as demonstrated by the decrease in glucose and lactic acid levels. In addition, the expression of metabolism-related enzymes involved in the Warburg effect (GLUT1, HK1, and LDHA) was suppressed by β-elemene. Collectedly, β-elemene attenuated the Warburg effect of NCI-H1650 cells via the miR-301a-3p/AMPKα axis.

## Discussion

**Figure 7:**
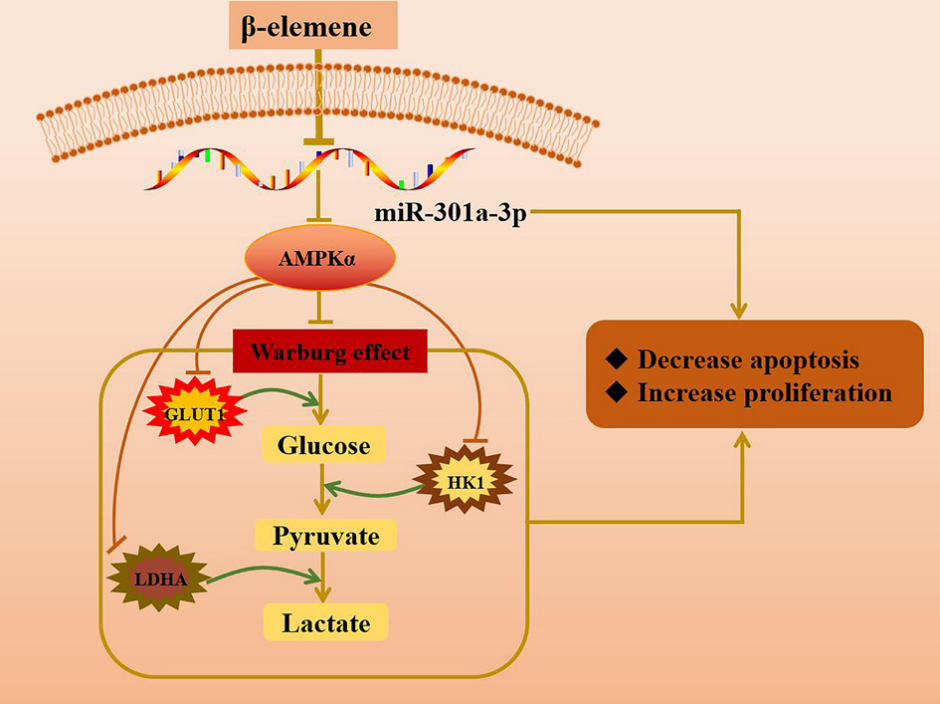
β-elemene attenuated the Warburg effect in NCI-H1650 cells through the miR-301a-3p/AMPKα axis β-elemene decreased the expression of miR-301a-3p and increased that of AMPKα, in turn inhibiting the Warburg effect in NCI-H1650 cells. This was demonstrated by the decrease in the levels of glucose uptake and lactic acid and the down-regulation of metabolism-related enzymes (GLUT1, HK1, and LDHA) involved in the Warburg effect.

This work presents evidence supporting that AMPKα was targeted by miR-301a-3p and that miR-301a-3p promoted the Warburg effect in NCI-H1650 cells via negative regulation of AMPKα. This was demonstrated by the increase in the levels of glucose and lactic acid, the up-regulation of metabolism-related enzymes (GLUT1, HK1, and LDHA) involved in the Warburg effect, and the decrease in AMPKα expression induced by miR-301a-3p mimics. These phenomena were reversed by AMPKα agonists. Furthermore, this work demonstrated that β-elemene attenuated the Warburg effect in NCI-H1650 cells through the miR-301a-3p/AMPKα axis (Figure 7).

As an anti-cancer drug, β-elemene combined with cisplatin showed remarkable anti-tumor efficiency in both clinically relevant patient-derived and cell-derived lung xenografts [18]. Zhao et al. reported that β-elemene is involved in inhibiting the growth of NSCLC cells via activation of AMPKα and ERK1/2 signaling [19]. Our preliminary experiments indicated that β-elemene increased the expression of AMPKα in A549 cells, thereby attenuating cell growth. However, the precise target and underlying molecular mechanism remain to be elucidated. MiR-301a-3p is an oncogenic miRNA involved in tumor metastasis, progression, and overall poor prognosis through different signaling pathways [11]. MiR-301a-3p overexpression accelerated colorectal cancer metastasis and proliferation via negative regulation of Runt-related transcription factor 3 and deleted in liver cancer-1 [20]. Microarray analysis suggested that miR-301a-3p was abnormally high expression in lung cancer [13]. However, the function of miR-301a-3p in lung cancer has not been extensively investigated. The present work demonstrated that miR-301a-3p promoted the growth of NSCLC cells. In addition, bioinformatics analysis suggested the existence of binding sites between AMPKα and miR-301a-3p. Inhibition of miR-301a-3p enhanced the expression of AMPKα and suppressed the Warburg effect. Thus, we hypothesized that the regulatory effect of β-elemene on AMPKα in NSCLC cells was associated with its modulatory effect on miR-301a-3p. This was verified by experiments showing that β-elemene decreased miR-301a-3p levels and increased the expression of AMPKα, in turn inhibiting the Warburg effect.

Instead of oxidative phosphorylation, cancer cells prefer to use glycolysis to provide energy for cell growth, and this process was identified as the Warburg effect. Evidence showed that AMPK was a negative regulator of aerobic glycolysis in cancer cells, and AMPKα deficiency promoted aerobic glycolysis as demonstrated by the increase in lactic acid production from glucose. In other words, down-regulation of AMPKα activity contributed to the Warburg effect in cancer cells [6]. The reactions of glycolysis are associated with GLUTs and rate-limiting enzymes such as HK1 and LDHA [21]. Increased production and membrane translocation of GLUTs dramatically promoted glucose uptake in tumorigenic cells [22]. LDHA is an important enzyme in aerobic glycolysis that catalyzes the conversion of pyruvate into lactate [23]. It was previously demonstrated that the up-regulation of GLUT1, LDHA, HK1/2, and pyruvate dehydrogenase-inhibitory enzymes pyruvate dehydrogenase kinase (PDK)-1 and PDK-4 induced the Warburg effect [24]. These supported the present conclusion that up-regulating the expression of AMPKα inhibited the Warburg effect of NCI-H1650 cells.

Collectively, this work demonstrated that miR-301a-3p increased the growth of NSCLC cells by regulating AMPKα-related Warburg effect, providing potential therapeutic targets for NSCLC. In addition, this work showed that β-elemene inhibited the Warburg effect by down-regulating the expression of GLUT1, HK1, and LDHA, which is meditated by the miR-301a-3p/AMPKα axis.

## Abbreviations

AMPK: adenosine monophosphate-activated protein kinase
cDNA: complementary DNA
FBS: fetal bovine serum
GAPDH: glyceraldehyde-3-phosphate dehydrogenase
GLUT1: glucose transporter 1
HK1: hexokinase 1
LDHA: lactate dehydrogenase A
MUT: mutant
NSCLC: non-small-cell lung cancer
PDK: pyruvate dehydrogenase kinase
PE: phycoerythrin
UTR: 3′-untranslated region
WT: wild-type
7-AAD: 7-aminoactinomycin D

## References

[1] Zheng F., Tang Q., Zheng X.H., Wu J., Huang H., Zhang H. et al. (2018) Inactivation of Stat3 and crosstalk of miRNA155-5p and FOXO3a contribute to the induction of IGFBP1 expression by beta-elemene in human lung cancer. Exp. Mol. Med. 50, 121 10.1038/s12276-018-0146-6 30209296 PMC6135838

[2] Bray F., Ferlay J., Soerjomataram I., Siegel R.L., Torre L.A. and Jemal A. (2018) Global cancer statistics 2018: GLOBOCAN estimates of incidence and mortality worldwide for 36 cancers in 185 countries. CA Cancer J. Clin. 68, 394–424 10.3322/caac.21492 30207593

[3] Siegel R.L., Miller K.D. and Jemal A. (2017) Cancer statistics, 2017. CA Cancer J. Clin. 67, 7–30 10.3322/caac.21387 28055103

[4] Unterlass J.E. and Curtin N.J. (2019) Warburg and Krebs and related effects in cancer. Expert Rev. Mol. Med. 21, e4 10.1017/erm.2019.4 31558177

[5] Herzig S. and Shaw R.J. (2018) AMPK: guardian of metabolism and mitochondrial homeostasis. Nat. Rev. Mol. Cell Biol. 19, 121–135 10.1038/nrm.2017.95 28974774 PMC5780224

[6] Faubert B., Boily G., Izreig S., Griss T., Samborska B., Dong Z. et al. (2013) AMPK is a negative regulator of the Warburg effect and suppresses tumor growth in vivo. Cell Metab. 17, 113–124 10.1016/j.cmet.2012.12.001 23274086 PMC3545102

[7] Zhao S., Wu J., Tang Q., Zheng F., Yang L., Chen Y. et al. (2016) Chinese herbal medicine Xiaoji decoction inhibited growth of lung cancer cells through AMPKalpha-mediated inhibition of Sp1 and DNA methyltransferase 1. J. Ethnopharmacol. 181, 172–181 10.1016/j.jep.2016.01.041 26850724

[8] Sun L., Liu X., Fu H., Zhou W. and Zhong D. (2016) 2-Deoxyglucose suppresses ERK phosphorylation in LKB1 and Ras wild-type non-small cell lung cancer cells. PLoS ONE 11, e0168793 10.1371/journal.pone.0168793 28033353 PMC5198974

[9] Guo J.C., Yang Y.J., Zhang J.Q., Guo M., Xiang L., Yu S.F. et al. (2019) microRNA-448 inhibits stemness maintenance and self-renewal of hepatocellular carcinoma stem cells through the MAGEA6-mediated AMPK signaling pathway. J. Cell. Physiol. 234, 23461–23474 10.1002/jcp.28915 31232474

[10] Xia X., Zhang K., Luo G., Cen G., Cao J., Huang K. et al. (2017) Downregulation of miR-301a-3p sensitizes pancreatic cancer cells to gemcitabine treatment via PTEN. Am. J. Transl. Res. 9, 1886–1895 28469793 PMC5411936

[11] Lettlova S., Brynychova V., Blecha J., Vrana D., Vondrusova M., Soucek P. et al. (2018) MiR-301a-3p suppresses estrogen signaling by directly inhibiting ESR1 in ERalpha positive breast cancer. Cell. Physiol. Biochem. 46, 2601–2615 10.1159/000489687 29763890

[12] Kara M., Yumrutas O., Ozcan O., Celik O.I., Bozgeyik E., Bozgeyik I. et al. (2015) Differential expressions of cancer-associated genes and their regulatory miRNAs in colorectal carcinoma. Gene 567, 81–86 10.1016/j.gene.2015.04.065 25925209

[13] Zhang Y., Sui J., Shen X., Li C., Yao W., Hong W. et al. (2017) Differential expression profiles of microRNAs as potential biomarkers for the early diagnosis of lung cancer. Oncol. Rep. 37, 3543–3553 10.3892/or.2017.5612 28498428

[14] Yu X., Xu M., Li N., Li Z., Li H., Shao S. et al. (2017) beta-elemene inhibits tumor-promoting effect of M2 macrophages in lung cancer. Biochem. Biophys. Res. Commun. 490, 514–520 10.1016/j.bbrc.2017.06.071 28624450

[15] Pan Y., Wang W., Huang S., Ni W., Wei Z., Cao Y. et al. (2019) Beta-elemene inhibits breast cancer metastasis through blocking pyruvate kinase M2 dimerization and nuclear translocation. J. Cell. Mol. Med. 23, 6846–6858 10.1111/jcmm.14568 31343107 PMC6787513

[16] Cai B., Ma L., Nong S., Wu Y., Guo X. and Pu J. (2018) Beta-elemene induced anticancer effect in bladder cancer through upregulation of PTEN and suppression of AKT phosphorylation. Oncol. Lett. 16, 6019–6025 30333873 10.3892/ol.2018.9401 PMC6176406

[17] Livak K.J. and Schmittgen T.D. (2001) Analysis of relative gene expression data using real-time quantitative PCR and the 2(-Delta Delta C(T)) Method. Methods 25, 402–408 10.1006/meth.2001.1262 11846609

[18] Cao M., Long M., Chen Q., Lu Y., Luo Q., Zhao Y. et al. (2019) Development of beta-elemene and cisplatin co-loaded liposomes for effective lung cancer therapy and evaluation in patient-derived tumor xenografts. Pharm. Res. 36, 121 10.1007/s11095-019-2656-x 31214786

[19] Zhao S., Wu J., Zheng F., Tang Q., Yang L., Li L. et al. (2015) beta-elemene inhibited expression of DNA methyltransferase 1 through activation of ERK1/2 and AMPKalpha signalling pathways in human lung cancer cells: the role of Sp1. J. Cell. Mol. Med. 19, 630–641 10.1111/jcmm.12476 25598321 PMC4369819

[20] Zhang L., Zhang Y., Zhu H., Sun X., Wang X., Wu P. et al. (2019) Overexpression of miR-301a-3p promotes colorectal cancer cell proliferation and metastasis by targeting deleted in liver cancer-1 and runt-related transcription factor 3. J. Cell. Biochem. 120, 6078–6089 10.1002/jcb.27894 30362160

[21] Yan L., Raj P., Yao W. and Ying H. (2019) Glucose metabolism in pancreatic cancer. Cancers (Basel) 11, 1460–1479 10.3390/cancers11101460 PMC6826406 31569510

[22] Lebelo M.T., Joubert A.M. and Visagie M.H. (2019) Warburg effect and its role in tumourigenesis. Arch. Pharm. Res. 10.1007/s12272-019-01185-2 31473944

[23] Pathria G., Scott D.A., Feng Y., Sang Lee J., Fujita Y., Zhang G. et al. (2018) Targeting the Warburg effect via LDHA inhibition engages ATF4 signaling for cancer cell survival. EMBO J. 37, 99735–99752 10.15252/embj.201899735 30209241 PMC6187221

[24] Yu W., Yang Z., Huang R., Min Z. and Ye M. (2019) SIRT6 promotes the Warburg effect of papillary thyroid cancer cell BCPAP through reactive oxygen species. Onco Targets Ther. 12, 2861–2868 10.2147/OTT.S194256 31114231 PMC6489652

